# A Brief Report on the Years Work and Progress of the Wesley House Clinic

**Published:** 1916-05

**Authors:** Clarence A. Rhodes

**Affiliations:** Atlanta, Ga.


### Journal-Record of Medicine

Successor to Atlanta Medical and Surgical Journal, Established 1855
and Southern Medical Record, Established 1870

**OWNED BY THE ATLANTA MEDICAL JOURNAL COMPANY**

PUBLISHED MONTHLY

*Official Organ Fulton County Medical Society, State Examing Board, Atlanta, Birmingham and Atlantic Railroad Surgeon's Association, Chattahoochee Valley Medical and Surgical Association, Etc.*

RICHARD R. DALY, M. D., Editor

A. W. STIRLING, M. D., C. M., D. P. H.; J. S. HURT, B. Ph., M.D.
GEO. M. NILES, M. D., W. J. LOVE, M. D., (Ala.); Associate Editors

E. W. ALLEN, Business Manager

**COLLABORATORS**

W. F. WESTMORELAND, M. D., General Surgery
F. W. McRAE, M. D., Abdominal Surgery
ALLEN H. BUNCE, Medical Societies
E. B. BLOCK, M. D., Diseases of the Nervous System
MICHAEL HOKE, M. D., Orthopedic Surgery
CYRUS W. STRICKLER, M. D., Legal Medicine and Medical Legislation
E. C. DAVIS, A. B., M. D., Obstetrics
E. G. JONES, A. B., M. D., Gynecology
ALLEN H. BUNCE, M. D., Pathology and Bacteriology
R. T. DORSEY, Jr., B. S., M. D., Medicine
L. M. GAINES, A. B., M. D., Internal Medicine
GEO. C. MIZELL, M. D., Diseases of the Stomach and Intestines
L. B. CLARKE, M. D., Pediatrics
EDGAR PAULLIN, M. D., Opsonic Medicine
THEODORE TOEPEL, M. D., Mechano Therapy
R. R. DALY, M. D., Diseases of the Eye, Ear, Nose and Throat
E. G. BALLENGER, M. D., Diseases of the Genito-Urinary Organs

VOL. LXIII. ATLANTA, GA., MAY, 1916. NO. 2

### A BRIEF REPORT ON THE YEARS WORK AND PROGRESS OF THE WESLEY HOUSE CLINIC.

By CLARENCE A. RHODES M. D., Atlanta, Ga.

About two years ago, I read to this Society, a paper entitled: "A Free Clinic, and Does it Pay?"

As this is a season in which great things are in store for the medical profession of Atlanta, we should not forget those things that have already been accomplished along the lines of free clinics and medical charities.

It is my purpose to set forth briefly what has been done by the Wesley House Clinics during the past year, taking into consideration the facilities and the equipment. Up to April, 1915, the clinics were confined to two rooms, viz: a reception room and one for general work, however, we have out-grown these quarters to such an extent, that more space was required, and to meet these exigencies, the officials of the Fulton Bag and Cotton Mills, came to our assistance most generously, meeting our requirements with both money and additional space. Through their liberality and aid, we are now occupying the second floor of a four-story structure for our clinical work, and we now challenge anyone to show us a better or more completely equipped clinic anywhere in the South, or one doing a better or greater work than that of The Wesley House.

Our present quarters are divided as follows: A general reception room, one side of which is the clinic and drug room, where examinations are made, treatments given and drugs dispensed. On the other side is the dental department, which was opened the first of the year, and is the Pride of the Wesley House, with six dentists in charge. The equipment is second to none, and the work executed is of the highest type. Just across the stairway is the nurse's private room, and adjoining this are two infirmary rooms with two beds each, with ample space for others if needed. Adjoining the infirmary rooms is the operating room with the surgeons' dressing room. The operating room is equipped for any and all kinds of operations; adjoining the operating room is the scrub and bath room with hot and cold water and other essentials; next comes the sterilizing room and the diet kitchen, equipped with the necessary apparatus and utensils.

The back bone of the clinic is Miss Mabel Wheeler, our most efficient nurse whose efforts are untiring and on whose shoulders rests the success of the entire medical organization. With such a nurse, and a staff of twenty-two of Atlanta's representative physicians and dentists, we indeed have a clinic which any community should feel justly proud·

As chief of staff, I would like to dwell at length as to the splendid work done in each department, but the success already attained stands out as a monument to the indefatigable energies and efforts of an able staff, hence, rather than over-step the time limit, I would much prefer that you hear personally from the gentlemen present who have contributed their time and talent toward the maintenance of an institution that lends succor to the sufferer.

To sumarize briefly, the following branches are covered with as little over-lapping as possible, viz: Medicine, Surgery, Orthopedic Surgery, Gynecology, Obstetrics, Eye, Ear, Nose and Throat, Pediatrics, Dermatology, Nervous and Mental Diseases, Pathology, X-Ray, Dentistry, Anaesthesia and Genito-Urinary Diseases.

The clinic is open for inspection at all times and we extend a cordial invitation to all physicians and especially to the Fulton County Medical Association to pay us a visit and see for themselves the smoothness and the wonderful influence this clinic is creating, and I believe, if this association, would familiarize themselves with the great amount of good we are doing, that it would lead to the establishment of similar clinical institutions in different parts of our city, that would be of untold benefit to humanity and to the community at large, thus adding greater fame to our fair city as a clinical center.

The Wesley House Clinic has long since passed the experimental stage, all obstacles have been overcome, and its continued growth and usefulness will be shown by future developments. The great Emory University may boast of its $1,000,000.00 endowment, but the Wesley House Clinic has besides Southern Methodism, the backing of a corporation, representing an investment of between $5,000,000 to $10,000,000. Hence with our able staff and the liberal co-operation and assistance we have received, our progress is assured.

The following will give some idea of the volume of work done by the clinic from January 1st, 1915, to and including December 31st, 1915:

Number of clinics held 244, an average of about five per week.

Number of patients treated at clinics 2,086, an average of nearly nine per clinic.

Visits made by nurse in the community 2.408, an average of 200 per month, or six per day.

Visits made in the community by the interne during eight months, 1,470, an average of 183 per month or six per day.

Prescriptions filled and dispensed 3,596, an average of 299 per month, or 10 per day.

Vaccinations 231, an average of 19 per month.

Office calls on the nurse 5,374, an average of 448 per month, or fifteen per day.

Dressings and treatments 2,133, an average of 175 per month, or six per day.

Patients sent to hospital 33. Obstetrical cases 21.

Operations with general anaesthesia 53, with local anaesthesia 43.

Number of families that were reached by clinics 500.

From May 1st to December 31, 1915, the following amount was collected by the clinic for medicines, dressings, etc. $112.15, the cost of same to the institution being $117·10; this insurred a shortage of $64.94.

During that period the average charge for prescriptions was five cents, but beginning with February 21st, 1916, we formulated new rules, whereby we hope to increase our income from this source in order to break even on medicines and dressings

The total donations to the clinic during the year has been estimated at $809.86, this does not include work done on the building, but supplies and equipment, etc.

In conclusion, as chief of staff and in behalf of the clinic, I desire to thank the following for their untiring efforts and willing support in making the clinic a success:

The ladies of the City Board of Missions.

The ladies of the Medical Committee who have worked faithfully in and out of season for the welfare of the clinic.

Our faithful nurse, Miss Mabel Wheeler, for her conscientious devotion to her multitudinous duties, her willingness and her capacity.

The members of the Medical and Dental Staffs for their efficient and faithful services and co-operation to the up-building of the clinic.

Mr. Oscar Elsas and Mr. Johnstone for their numerous useful suggestions and their liberal assistance in equipping and housing the clinic that must necessarily redound to their credit.

